# Comparison of spatiotemporal patterns of historic natural Anthrax outbreaks in Minnesota and Kazakhstan

**DOI:** 10.1371/journal.pone.0217144

**Published:** 2019-05-17

**Authors:** Kaushi S. T. Kanankege, Sarsenbay K. Abdrakhmanov, Julio Alvarez, Linda Glaser, Jeffrey B. Bender, Yersyn Y. Mukhanbetkaliyev, Fedor I. Korennoy, Ablaikhan S. Kadyrov, Aruzhan S. Abdrakhmanova, Andres M. Perez

**Affiliations:** 1 Department of Veterinary Population Medicine, College of Veterinary Medicine, University of Minnesota, St. Paul, Minnesota, United States of America; 2 S. Seifullin Kazakh Agrotechnical University, Astana, Kazakhstan; 3 Centro de Vigilancia Sanitaria Veterinaria (VISAVET), Departamento de Sanidad Animal, Facultad de Veterinaria, Universidad Complutense, Madrid, Spain; 4 Minnesota Board of Animal Health, St. Paul, Minnesota, United States of America; 5 Environmental Health Sciences, School of Public Health, University of Minnesota, Minneapolis, Minnesota, United States of America; 6 FGBI Federal Center for Animal Health, mkr. Yurevets, Vladimir, Russia; Spectrum Health, UNITED STATES

## Abstract

Disease spread in populations is a consequence of the interaction between host, pathogen, and environment, i.e. the epidemiological triad. Yet the influences of each triad component may vary dramatically for different settings. Comparison of environmental, demographic, socio-economic, and historical backgrounds may support tailoring site-specific control measures. Because of the long-term survival of *Bacillus anthracis*, Anthrax is a suitable example for studying the influence of triad components in different endemic settings. We compared the spatiotemporal patterns of historic animal Anthrax records in two endemic areas, located at northern latitudes in the western and eastern hemispheres. Our goal was to compare the spatiotemporal patterns in Anthrax progression, intensity, direction, and recurrence (disease hot spots), in relation to epidemiological factors and potential trigger events. Reported animal cases in Minnesota, USA (n = 289 cases between 1912 and 2014) and Kazakhstan (n = 3,997 cases between 1933 and 2014) were analyzed using the spatiotemporal directionality test and the spatial scan statistic. Over the last century Anthrax occurrence in Minnesota was sporadic whereas Kazakhstan experienced a long-term epidemic. Nevertheless, the seasonality was comparable between sites, with a peak in August. Declining number of cases at both sites was attributed to vaccination and control measures. The spatiotemporal directionality test detected a relative northeastern directionality in disease spread for long-term trends in Minnesota, whereas a southwestern directionality was observed in Kazakhstan. In terms of recurrence, the maximum timespans between cases at the same location were 55 and 60 years for Minnesota and Kazakhstan, respectively. Disease hotspots were recognized in both settings, with spatially overlapping clusters years apart. Distribution of the spatiotemporal cluster radii between study sites supported suggestion of site-specific control zones. Spatiotemporal patterns of Anthrax occurrence in both endemic regions were attributed to multiple potential trigger events including major river floods, changes in land use, agriculture, and susceptible livestock populations. Results here help to understand the long-term epidemiological dynamics of Anthrax while providing suggestions to the design and implementation of prevention and control programs, in endemic settings.

## Introduction

Anthrax is a zoonotic disease affecting livestock, wildlife, and humans which caused by the spore-forming bacterium *Bacillus anthracis* [1]. Anthrax spores survive for extended periods of time in the environment and rapidly return to vegetative stages once conditions are favorable [2]. Therefore, recurrence of Anthrax is observed in endemic areas years apart [2, 3], which makes the recognition of risk areas and control zones a challenging task. Epidemiological determinants of Anthrax include soil pH, calcium, and organic matter content [4], precipitation, temperature, wind, and vegetation biomass [5–7]. Additionally, river drainages/flood plains, wild herbivore population, human population densities, and anthropogenic activities, such as limestone mining or road building, are recognized to play a role in the occurrence of Anthrax outbreaks [1, 8].

Recurrence of Anthrax in endemic areas is often analyzed and modeled in relation to the environmental characteristics of the setting, to identify high risk areas [6, 7], despite differences in the demographic, socio-economic, and historical backgrounds. However, a recent study which attempted to predict habitat suitability for *Bacillus anthracis* in Kazakhstan using an ecological niche model (ENM) fitted to USA data (and vice versa), failed to accurately predict the pathogen presence in novel landscapes [9]. Their observed differences in ENM predictions were attributed to the genetic-ecological divergence of the pathogen at different locations, suggesting that site-specific features should be considered when assessing the disease epidemiology and, ultimately, planning for prevention and control activities. Additionally, one may argue that it is equally important to understand the influence of spatiotemporal epidemiological factors and trigger events that led to the Anthrax occurrences, in addition to the recognition of ecological suitability. The inability to accurately predict the pathogen presence/suitability in novel landscape using ENM, as seen in Mullins et al. [9] study, potentially is explained by the differences and dynamics of the demographic, socio-economic, and agricultural-livestock production backgrounds in different settings, which play a major role in determining the spread of pathogens.

As suggested by the epidemiological triad concept, the interaction between host, pathogen, and environment results in disease patterns of populations. Yet the influences of each triad component may dramatically vary for different spatiotemporal conditions. The spatiotemporal characterization of historic Anthrax occurrence would facilitate identifying the patterns of climatic, anthropogenic, socioeconomic, agricultural, and environmental changes that may have led to the relevant disease outbreaks. Understanding those epidemiological factors and trigger events which may have influenced the spread of Anthrax, especially at the hotspots in which Anthrax recurs, may support suggesting tailored and site-specific risk zones to improve planning of control measures.

The site-specific spatiotemporal patterns of Anthrax spread could be better demonstrated in a generalizable manner if more than one endemic site was assessed. Here, two distinctly different Anthrax endemic sites, located at similar latitudes in the eastern and western hemispheres were selected for this study, namely, the U.S. state of Minnesota (latitudes 44° through 48°, North) and the Republic of Kazakhstan (latitudes 42° through 56°, North). Moreover, the socio-economic and demographic conditions of the two settings have been substantially different, historically. Both Minnesota and Kazakhstan have recorded Anthrax cases since early 1900’s [7, 10]. Although ENM approaches were used to compare between the two sites [9], the historic spatiotemporal patterns of both sites have not been analyzed retrospectively to identify potential disease hotspots and the trigger events.

The objectives of this study were to understand and compare the progression, intensity, direction, and recurrence of animal Anthrax cases in relation to epidemiological factors and trigger events in Minnesota and Kazakhstan over an extended period of time. The analysis was intended to generate hypotheses regarding the association of the outbreaks with site specific trigger events, to ultimately suggest control zone boundaries based on the reported data.

## Methods

### Data

The case definition encompasses locations where one or several species of animals were reported to be dead or affected by Anthrax, including both suspected and laboratory confirmed animals, during the period of data collection. In Minnesota, 289 animal Anthrax cases reported between 1912 and 2014 to the Minnesota Board of Animal Health were used in the analysis. For events occurred between 1920 and 1999 the geographical coordinates were obtained using historic aerial images whereas, for those cases that occurred after 2000, coordinates were recorded during site visits [10]. In addition to location details, the database contained the number and species affected and date in which the disease was first reported. Both laboratory confirmed (n = 233; 80.6%) and suspected cases based on clinical presentation were included in the analysis [10].

For the Republic of Kazakhstan, cases reported by the Cadastral register of stationary unfavorable foci on Anthrax between 1933 and 2014 (n = 3,997) were analyzed. Majority of the cases prior to 1940’s were suspected cases based on clinical presentation and epidemiological characteristics. Kazakhstan’s database contained coordinates of the location, year, number, animal species affected, and for 2,027 (53.6%) cases, the date of report.

### Analysis

A descriptive analysis was performed to characterize the animal species affected and the locations with recurrent Anthrax cases. Epidemic curves were used to visualize the annual progression of Anthrax cases. Seasonality of animal Anthrax occurrence was explored by plotting the monthly sum of cases in each location. Although all records contained the year of the case, the precise date of report was only available for 53.6% of the records from Kazakhstan. Therefore, in the descriptive analysis on seasonality, the remaining 1,970 records in which only the year was provided were eliminated. Additionally, the G-rates of animal Anthrax, i.e. the number of cases over a land area unit in one year [11], were calculated to compare the heterogeneity of disease occurrence of both sites.

Spatiotemporal directionality of Anthrax spread was analyzed using the spatiotemporal directionality test implemented in the ClusterSeer v.2.05. software (User manual, 2012: https://www.biomedware.com/). The spatiotemporal directionality test was used to detect the space-time interaction through a calculation of the mean direction of Anthrax spread. In this analysis, the directionality is measured by means of the angle, i.e. the rotation from the horizontal axis (with east corresponding to 0°), and the angular concentration (AC). The AC quantifies the magnitude of the variance in the angles between vectors connecting pairs of cases (value between 0 and 1), where the closer the values get to one, the higher the consistency in the direction of disease spread. A relative matrix was computed for the analysis, which uses vectors connecting each case to all the following cases to measure the directionality. The significance of the mean direction was evaluated through a randomization procedure, which retains constant geographic locations and randomly assigns the time between pairs of cases. The p-value was determined by comparing the AC from the original (non-randomized) data to the null distribution generated through the randomization process in 999 iterations (User manual, 2012: https://www.biomedware.com/).

The permutation model of the scan statistic was used to quantify the spatiotemporal pattern of disease occurrence in both settings, with the intention to identify areas with recurrent outbreaks (disease hot spots) [12]. The spatiotemporal permutation model creates cylinders of candidate clusters of disease throughout the study area and time period. The base and height of the cylinder, representing space and time respectively, varies up to a maximum value of 50% of the study population [12], which determines the possible maximum size of the cluster. The null hypothesis of the permutation model of the scan statistic is based on an even distribution of cases in time and space regardless of the distribution of controls or the population at risk. The ratio between observed and expected number of cases within each candidate cylinder is computed and the significance of the cluster is tested using a Monte Carlo simulation process in which time labels are randomly assigned to each location 999 times [12]. Candidate clusters with a P ≤0.05 were assumed to represent space-time clusters at high risk for occurrence of the disease. The test was implemented using the SaTScan software (http://www.satscan.org/ version 9.6). The spatiotemporal permutation model of the spatial scan statistic was set to a maximum of 10% and 5% for the spatial and time windows, respectively. The influence of this choice of space-time parameters was assessed through a sensitivity analysis by repeating the cluster analysis with maximum spatial window sizes of 5%, 10%, 20%, 30%, and 50% while the maximum temporal window sizes were held at 5% and 10% (S1 Fig). We defined an outbreak, in an Anthrax endemic setting, as a statistically significant spatiotemporal aggregation of animal Anthrax spread beyond 1km radius within a maximum of 3-year time span.

## Results

**Fig 1:**
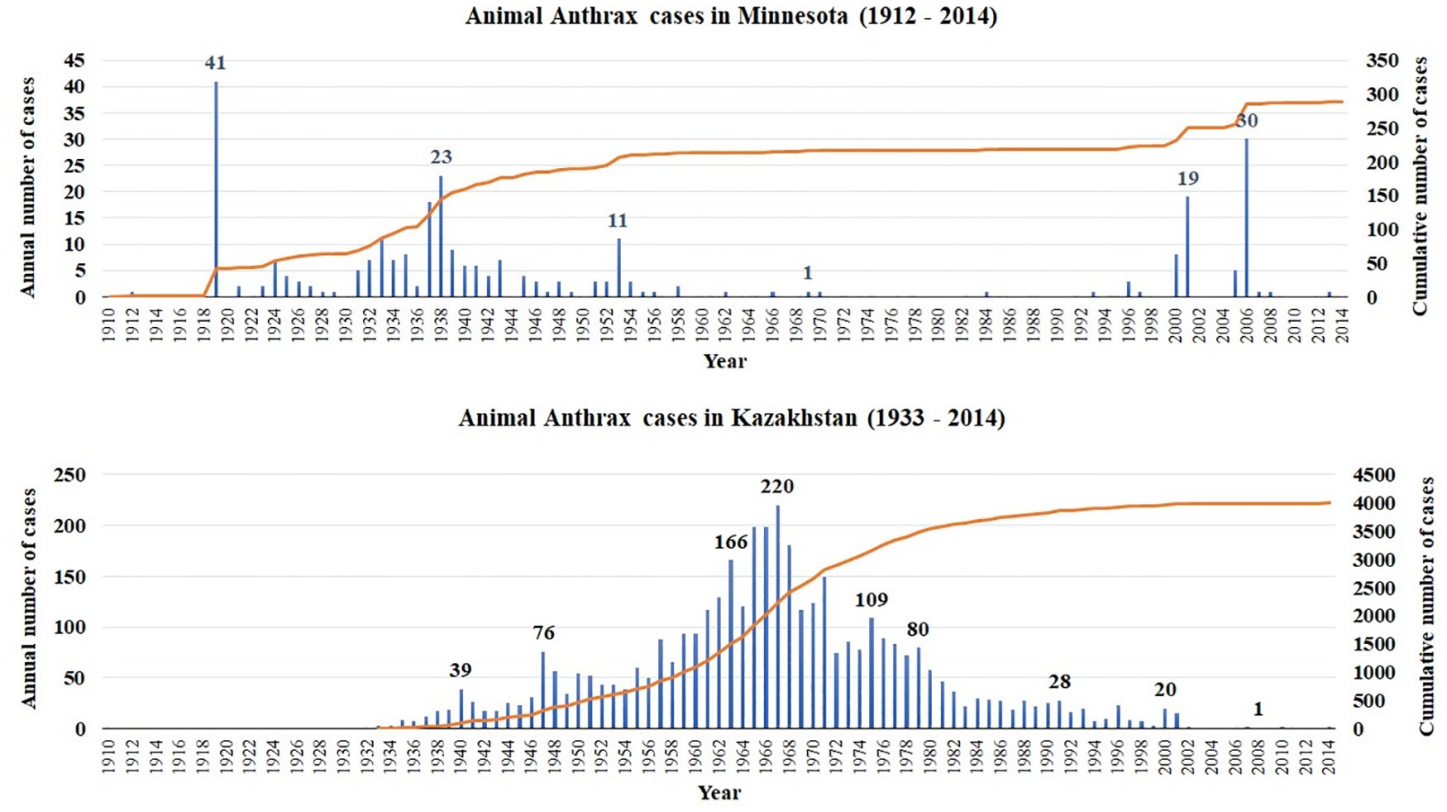
Epidemic curves illustrating the number of animal Anthrax outbreaks. Outbreaks were reported between 1912 and 2014 in Minnesota and between 1933 and 2014 in Kazakhstan (Bars indicate annual number of reported cases, left axis, and lines indicate cumulative number of cases, right axis).

**Fig 2:**
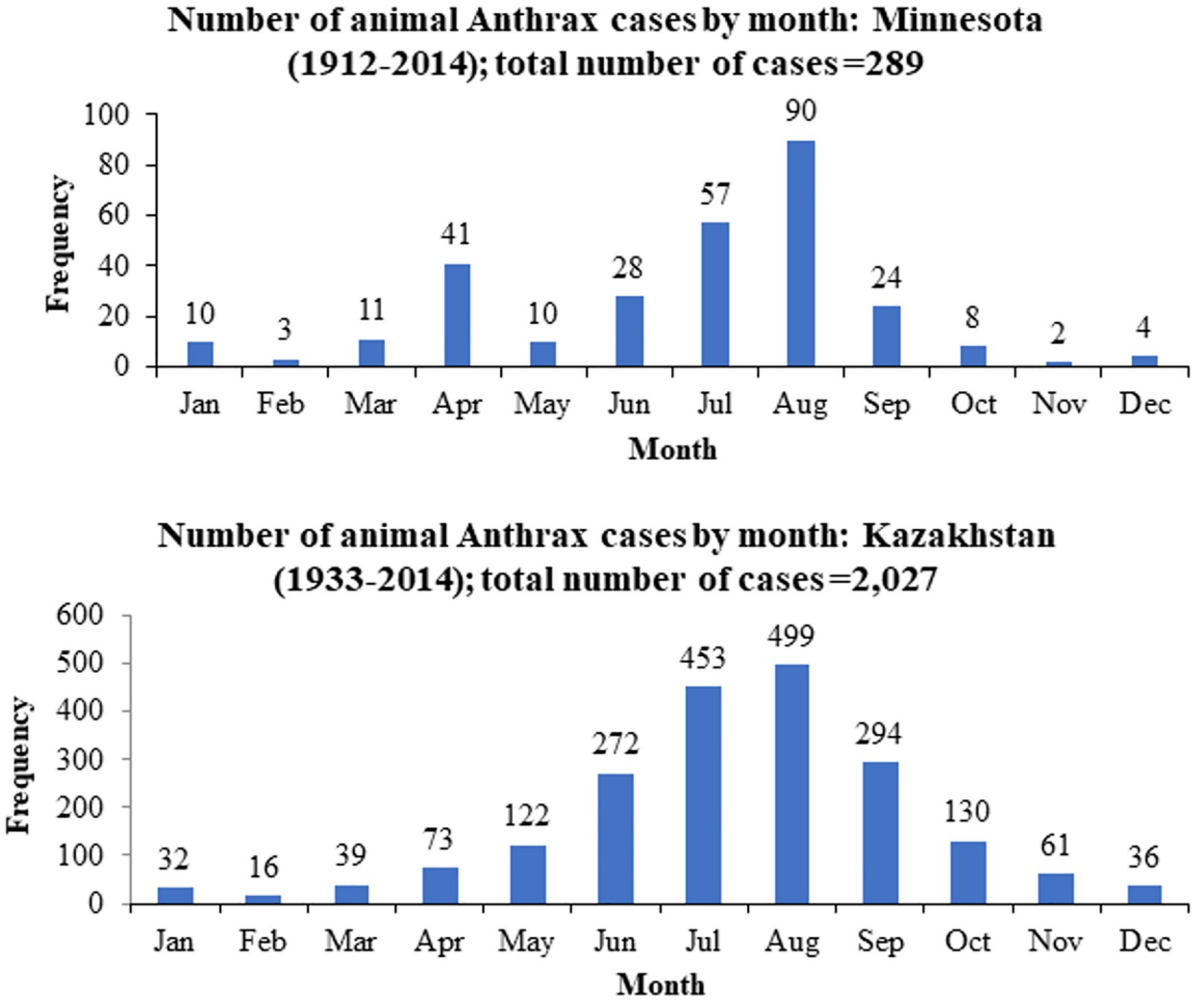
Number of animal Anthrax cases by month in Minnesota (1912–2014) and Kazakhstan (1933–2014). The number of cases included in the monthly analysis (n) were 289 and 2,027 for Minnesota and Kazakhstan (53.6% of the total records of Kazakhstan) respectively.

Minnesota experienced a period of multiple outbreaks prior to 1958, with three peaks in 1919, 1938, and 1953, respectively (Fig 1). After 1958, the number of cases remained minimum for nearly 4-decades and two major outbreaks were reported again in years 2000 and 2005 (Fig 1). The epidemic curve of Kazakhstan was consistent with a prominent and lengthy epidemic of animal anthrax that began in the 1940’s with a peak of 220 cases reported in 1968, and decreased after 2000 (Fig 1). Most of the cases in both Minnesota and Kazakhstan were reported in summer months, with a peak in August (Minnesota with n = 90/289; 31.1% and Kazakhstan with n = 499/3,229; 24.6% respectively) (Fig 2).

**Fig 3:**
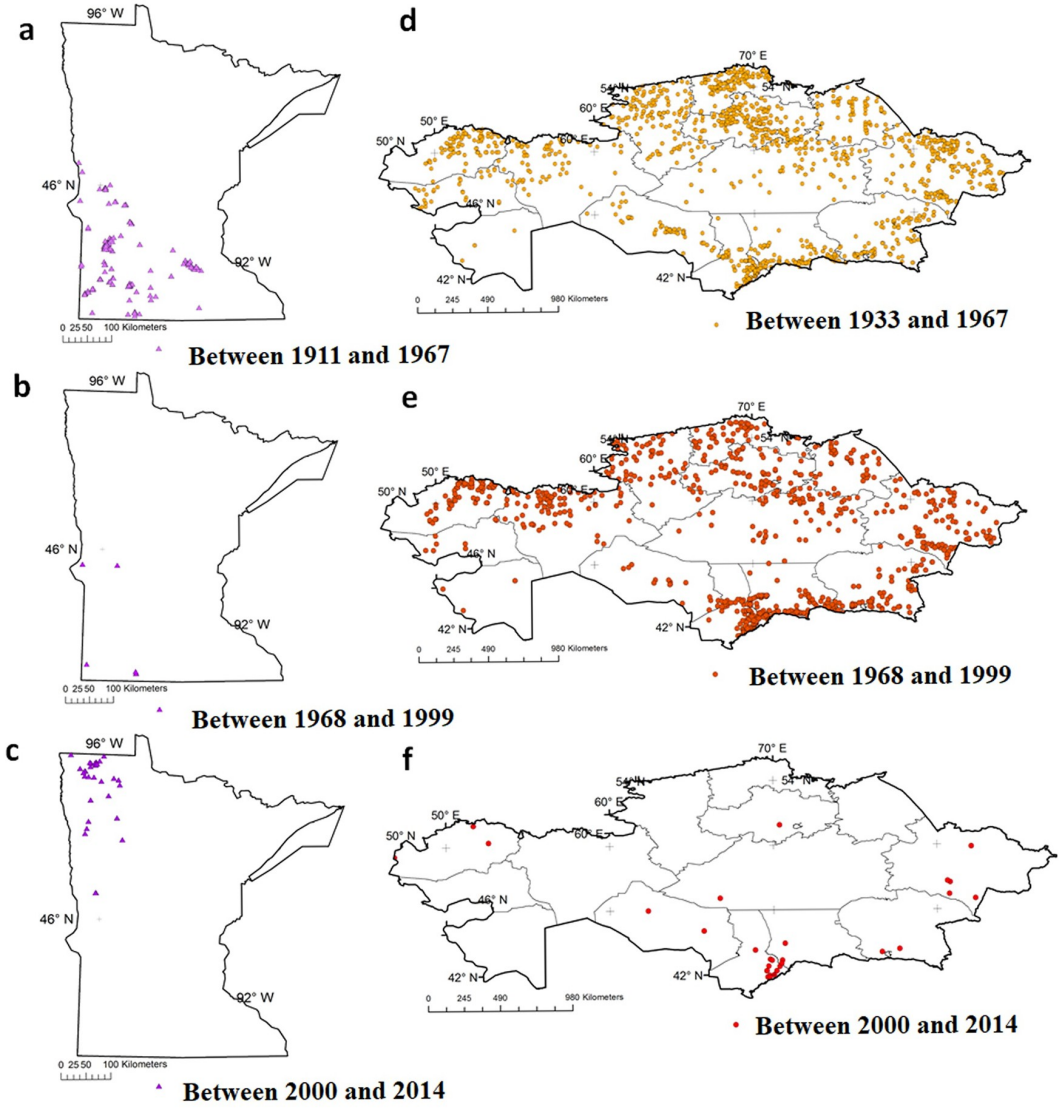
Maps illustrating the distribution of animal Anthrax at certain time intervals. Panels a, b, and c represents cases in Minnesota between 1912 and 2014. Panels d, e, and, f represents cases in Kazakhstan between 1933 and 2014.

Anthrax cases were not homogenously spread across the geographical space in Minnesota or Kazakhstan before and after the most recent/peak outbreak (Figs 1 and 3). In Minnesota, a total of 222 unique locations with Anthrax cases were reported between 1912 and 2014. Most of the locations affected before the year 2000 were located in the southwestern part of Minnesota, whereas most of the cases reported after 2000 were located in the north-western corner of the State (Fig 3). A total of 209 cases occurred in cattle, 119 in horses, 100 in swine, 55 in canids, and 9 in small ruminants. Some (n = 28) locations/farms reported recurrence of the disease ≥2 years apart, with a mean, median, and maximum time-spans of 10.9, 7, and 55 years (1941 and 1996) between recurrent cases in the same location.

In Kazakhstan, a total of 1,797 unique locations with animal Anthrax cases were reported between 1933 and 2014. The number of cases by animal species affected in Kazakhstan were 2,398 cattle, 1,210 small ruminants, 225 equine, 152 swine, 6 camels, 5 canids (dogs and foxes), and one mink. Among the 1,797 locations/farms, there were 970 reported recurrences ≥2 years apart, with a mean, median, and maximum time-span of 7.47, 5, and 60 years (1947 and 2007) between recurrent cases in the same location, respectively.

According to the G-rate indicator of heterogeneity, compared to Minnesota (222 total cases in a 225,180 km^2^ area, G-rate = 0.012±0.02), the overall number of cases reported from Kazakhstan (3,997 total cases in a 2.725+06 km^2^ area, G-rate 0.0178±0.019) was relatively high (unit of G-rate: number of cases per 1000 km^2^ within a year).

According to the spatiotemporal directionality test performed using the relative time matrix, a northeastern directionality (81°) was found in Minnesota and a southwestern directionality for Kazakhstan (260°) with low (~0.1) though significant (p<0.05) AC values in both locations.

**Fig 4:**
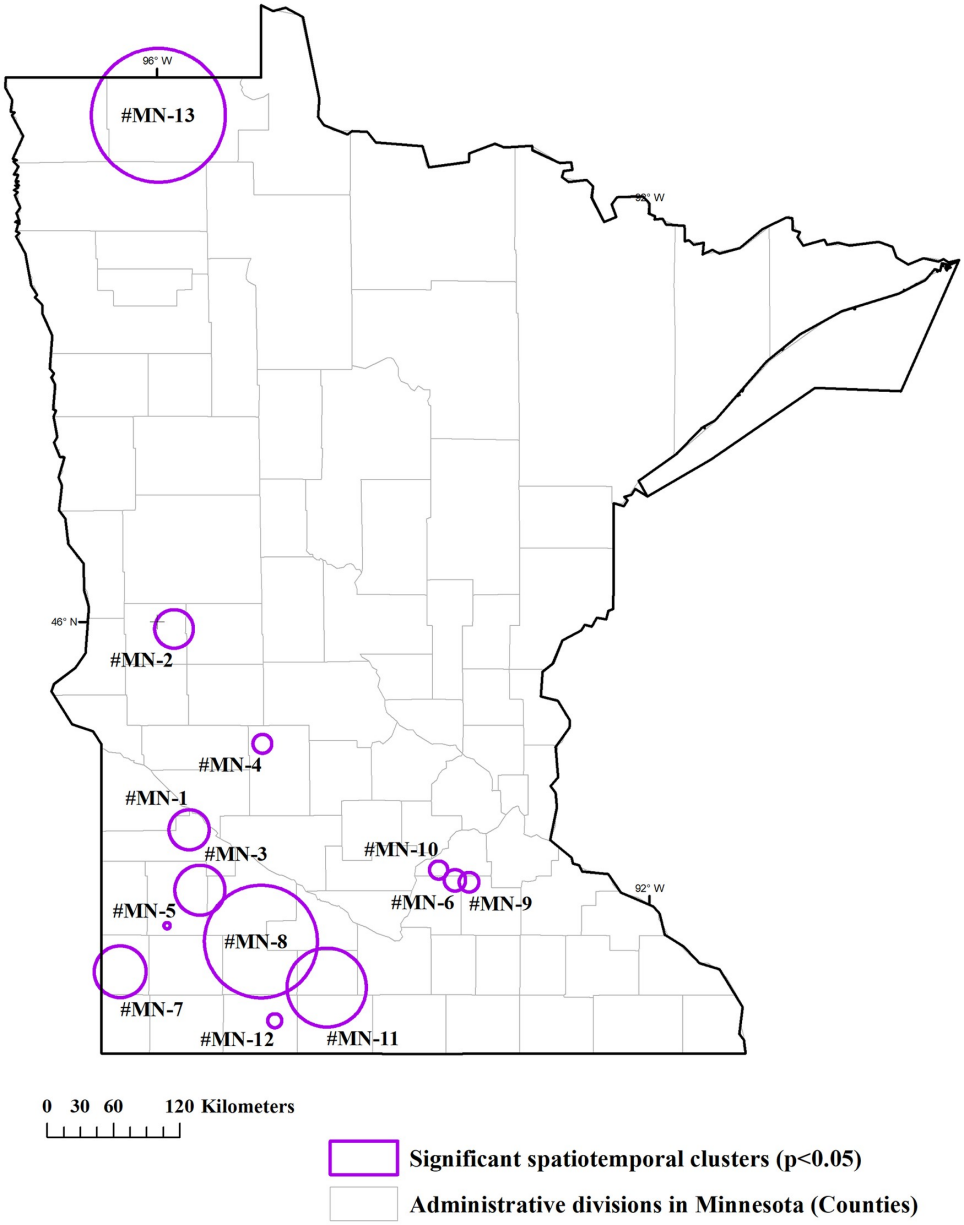
Spatiotemporal clustering of animal Anthrax cases in Minnesota (time: 1912–2014). Clusters were detected using the space-time permutation model of the spatial scan statistic with the spatial-window set to 10% and 5%-time window. Clusters are numbered according to the order of the start date of the outbreak.

**Fig 5:**
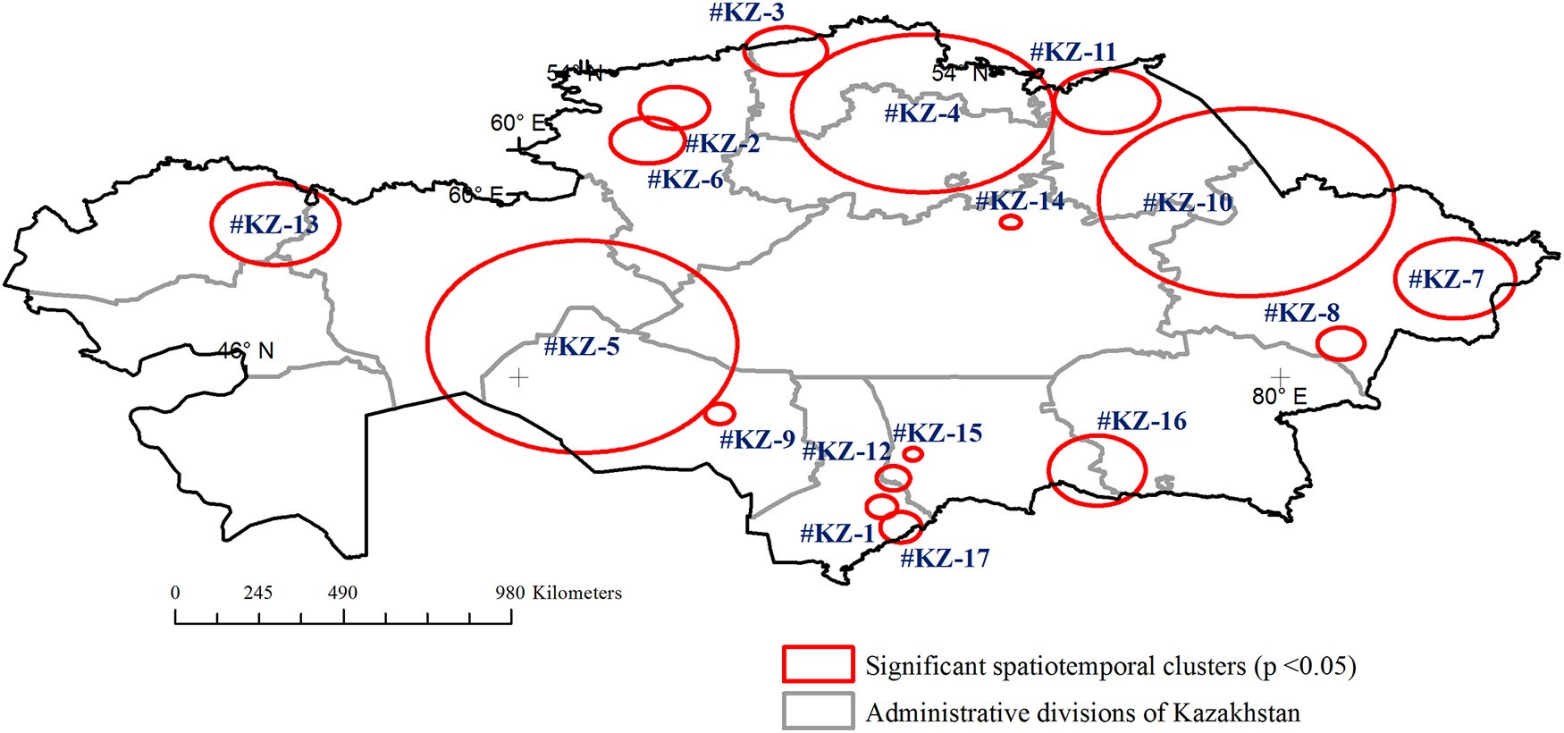
Spatiotemporal clustering of animal Anthrax cases in Kazakhstan (time: 1933–2014). Clusters were detected using the space-time permutation model of the spatial scan statistic with the spatial- and time-windows set to 10% and 5%, respectively. Clusters are numbered according to the order of the start date of the outbreak.

The spatiotemporal permutation model of the scan statistic detected 13 significant (p<0.05) clusters in Minnesota with spatial and temporal sizes spanning between 2 and 80 km and 1 to 2 years and observed-to- expected ratios (O/E) ranging between 4 and 63 (Fig 4 and Table 1). There was a location where Anthrax was first reported in 1941 and then again in 1996, i.e. 55-years apart. When the recurrence occurred in 1996 this area resulted in an outbreak, as indicated by cluster #MN-12. In Kazakhstan 17 significant (p<0.05) spatiotemporal clusters with radii ranging between 18 and 309 km and temporal lengths between1 to 3 years, and O/E between 3 and 57 were obtained (p<0.05) (Fig 5 and Table 1). The location where Anthrax was reported 60 years apart, i.e. first in 1947 and again in 2007, was located within the area captured by cluster #KZ-4, towards the west of Astana, Kazakhstan. When the spatiotemporal cluster radii were compared between the sites (Table 1), relatively smaller spatiotemporal clusters ranging between 2.07 to 39.9 km (median = 12.1 km and average = 14.8 km) were found for Minnesota, whereas, Kazakhstan’s clusters ranged between 18.4 to 309.2 km (median = 66.6 km and average = 98.0 km) (S2 Fig).

**Table 1:** Summary of spatiotemporal clusters of Anthrax detected in Minnesota (between 1912 and 2014) and Kazakhstan (between 1933 and 2014), using the spatiotemporal permutation model of the scan statistics. The spatial and temporal windows of the scan statistic were set to 10% and 5% respectively.

Cluster ID	Radius (km)	Number of case locations within the cluster	Start (Year)	End (Year)	Observed-to-expected ratio (O/E)
**Minnesota**					
#MN-1	12.88	22	1919	1919	5.72
#MN-2	12.14	8	1923	1924	28.44
#MN-3	16.16	5	1925	1926	29.39
#MN-4	5.99	8	1931	1932	19.20
#MN-5	2.07	5	1932	1933	16.00
#MN-6	6.91	6	1933	1933	12.08
#MN-7	16.89	13	1937	1938	5.07
#MN-8	36.61	13	1938	1939	6.16
#MN-9	6.36	6	1945	1946	27.43
#MN-10	5.88	3	1948	1948	72.00
#MN-11	25.80	4	1953	1953	26.18
#MN-12	4.59	4	1996	1997	48.00
#MN-13	39.87	14	2001	2001	10.61

**Kazakhstan**					
#KZ-1	32.94	18	1934	1937	10.77
#KZ-2	61.04	15	1935	1938	34.48
#KZ-3	69.90	24	1940	1940	57.41
#KZ-4	229.99	270	1944	1947	5.38
#KZ-5	309.19	38	1945	1947	7.70
#KZ-6	66.65	15	1949	1952	9.50
#KZ-7	115.82	43	1951	1954	4.59
#KZ-8	46.97	23	1954	1957	4.89
#KZ-9	29.86	6	1956	1958	10.65
#KZ-10	273.36	157	1956	1959	2.70
#KZ-11	91.66	36	1968	1969	5.61
#KZ-12	35.48	10	1971	1971	13.09
#KZ-13	119.16	42	1981	1983	8.65
#KZ-14	18.67	4	1982	1983	41.98
#KZ-15	18.40	7	1985	1987	35.13
#KZ-16	102.38	51	1989	1992	5.25
#KZ-17	44.72	36	2000	2001	7.81

The sensitivity analysis (S1 Fig) indicated that the parameter choice of spatial window of 10% and temporal window of 5% comply with the outbreak definition. When used 5% space and 5% time window, three separate clusters were detected in northwestern Minnesota: one cluster in 2001 and two in 2006. However, in Kazakhstan, with the 5% space and 5%time window parameters, two clusters with < 1 km radii were detected in the southeastern region of the country in year 2001. Except for the additional clusters, the locations of clusters detected by 10% and 5% space-time combination was comparable with the 5% and 5% space-time combination.

## Discussion

The study compared historic animal Anthrax occurrence in two endemic areas at similar northern latitudes in the western and eastern hemispheres, where socio-economic and demographic conditions were drastically different, with the objective of comparing spatiotemporal patterns of disease progression (epidemic curves), intensity (G-statistics), direction (spatiotemporal directionality test), and recurrence (disease hot spot detection using spatiotemporal cluster analysis) in relation to potential trigger events. We expect that the analysis here would be useful when suggesting control zone boundaries for each site, based on the reported data.

The progression and intensity of Anthrax was site-specific with few comparable characteristics between the assessed settings; sporadic Anthrax outbreaks were observed in Minnesota, whereas a long-term epidemic was reported in Kazakhstan. This long-term epidemic in Kazakhstan may be attributable to the absence and inadequacies of mass animal vaccination during early years [7, 13]. According to G-rate indicator values of the heterogeneity, in Kazakhstan it was 1.5 times higher than in Minnesota (i.e. the difference of G-rates is 0.0178–0.012 = 0.0066). This difference in heterogeneity further indicates the requirement of mass vaccination and control strategies in Kazakhstan compared to Minnesota. In alignment with the literature from the northern hemisphere [14], the majority of the outbreaks were reported during the summer months in the regions with peaks in August (Fig 2), suggesting an association with the practice of livestock grazing during summer (approx. May through end of September). The summer grazing practice, especially before the mid-20^th^ century, coincides with the potential exposure to pasture/soil-contaminated with pathogens [15, 16].

The relative directionality of long-term trends in Anthrax cases in Minnesota suggested a northeastern directionality of Anthrax compared to the early cases of the disease, which were reported in the south-west of Minnesota. There was a notable change in the geographical area in which Anthrax cases were reported in the State after 1999 (Fig 3). The early Anthrax cases were reported from southwestern Minnesota, in the Minnesota River valley, whereas the outbreaks reported between 2000 and 2014 were reported from the northwestern corner of the state, in the Red River valley. This northwestern trend may involve multiple trigger events during the period immediately before the year 2000 including the Red River flooding of 1997 [17], Anthrax outbreaks of North Dakota in 1998 [18], and the potential wildlife behavioral and migration patterns in the Northern Great Plains including Bison and other habitat sharing ungulates (Elk and White tailed deer) [19, 20, 21]. Whereas, in Kazakhstan, the relative time measure of the spatiotemporal directionality test identified a southwestern directionality indicating higher potential of Anthrax in the southwestern area in the last decades compared to the past. However, Anthrax occurrence in Kazakhstan was observed throughout the country since early years which is potentially attributable to seasonal movements of livestock with nomadic migrations [22].

The recurrence patterns of Anthrax were comparable between the sites. The maximum time-span of anthrax recurrence at the same location in Minnesota and Kazakhstan was 55 and 60 years respectively. *B*. *anthracis* spores have a high environmental resistance under certain environmental conditions such as depth and organic content of contaminated soil, and a half-life of nearly 100 years has been reported [23]. The active lifecycle of *B*. *anthracis* in soil involving bacteriophage-mediated ecological adaptations also suggested to play a role [24]. Occurrence of cases in the same locations could be therefore due to disturbance of buried carcasses containing viable spores but also could be a result of recurrent recontamination of the soil due to unappreciated deaths of anthrax-infected animals [14, 25].

Among the 13 spatiotemporal clusters detected in Minnesota, six were spatially overlapping, indicating potential hot spot areas: Clusters #MN-6, 9, and 10 and Clusters #MN-3, 8, and 11. The major trigger events/factors that are likely contributing to Anthrax outbreaks in Minnesota include: 1) favorable soils in western and northwestern Minnesota [6], 2) historic river flooding events in the Minnesota and Red rivers that contribute to spread of pathogens and spores to extensive areas [17], and 3) changes in distribution of susceptible livestock populations and management including extensive beef cattle production during early 1900’s in the southern prairies of Minnesota and summer grazing [15, 26] (S1 Table). The declining trend of recent cases in Minnesota may be attributable to direct and indirect preventive measures including: 1) flood control programs implemented targeting Minnesota and Red river areas [27, 28]; 2) prompt vaccination [29]; and reduced summer grazing and increased feedlot management practice during late 2000’s [15].

There were 17 spatiotemporal clusters in Kazakhstan, among which eight were overlapping indicating potential hot spot areas: #KZ-1, 12, 15, and 17; #KZ-2 and 6; and #KZ-5 and 9. The major trigger events/factors that are likely contributors to Anthrax outbreaks in Kazakhstan include: 1) favorable soils [7]; 2) insufficient vaccination coverage or absence of vaccination [7]; 3) livestock growth since 1934 (National Committee of Statistics (In Russian): http://istmat.info/node/21348) including the influx of inhabitants along with their livestock from other USSR regions [30–32]; 4) seasonal movements of livestock with nomadic migrations, historically [22]; 5) land use, especially the Virgin Lands Program which lead to disturbance of previously unused landscapes where some of the previous Anthrax burial sites were located, which may have resulted in bringing the anthrax spores onto the surface and its intensive wind mediated spread over long distances [5, 22, 31, 32]; 6) river flooding, especially the Syrdarya river flooding which might be related to disturbances at old carcass burial sites [13, 33]; 7) range-land based livestock production [13, 34] where susceptible populations may come in contact with the contaminated soils/pasture; and 8) intense agriculture in the southern Kazakhstan where high human population density, intensive agriculture, together with livestock population collectively may have resulted in frequent disturbance of anthrax burials and animal contacts with the pathogen [30] (S2 Table). The declining trend of the recent cases in Kazakhstan may be attributable to direct and indirect preventive measures including: mass vaccination and mandatory disposal by burning carcasses of animals that had died from anthrax [35]; 2) development and implementation of a whole complex of preventive, antiepizootic and antiepidemic measures, continuous epizootic monitoring and recording of anthrax burial sites [13].

The spatiotemporal cluster radii may be useful when understanding the potential extent of Anthrax spread as well as determining control and surveillance zones in endemic settings. Based on the distribution of spatiotemporal cluster radii (Table 1 and S1 Fig), we suggest that the surveillance or control zones for Minnesota may consider a range of 2 to 40 km with a median of 12 km for preventive activities such as vaccination. As per the current recommendations of Minnesota Board of Animal Health, spring vaccination is recommended in the Northwestern part of the state of Minnesota. Once a confirmed case is reported, the recommendations include: vaccination and administration of antibiotics as needed to safeguard exposed animals in the herd, notification and recommendation for vaccination of susceptible animals within 10 miles (~16 km) and recommendation to monitor the herds within 10 to 30 miles (16 to 48 km). Further details regarding the vaccine recommendations for Midwestern USA and adjacent Canadian states, i.e. The Unified Anthrax Recommendations, are found elsewhere [36]. Vaccine recommendations are suggested considering the potential underreporting or delayed reporting scenarios where contamination of Tabanid fly mouth parts with the pathogen and thereby transport pathogen to unvaccinated susceptible animals on neighboring areas [36]. Similarly, the ideal surveillance or control zones for Kazakhstan may have a range between 18 to 310 km, with a median of 67 km. The previous recommendations against animal Anthrax in Kazakhstan involved mass vaccination of all the susceptible animals since 1961 and bi-annual vaccination since 1982 [37]. Anthrax is a priority disease in Kazakhstan under the National Veterinary Legislation, and with the introduction of improved vaccines which provide year-long immunity [38, 39], the current regulations demand mandatory annual re-vaccination.

The limitations of this study include: the use of officially reported data (therefore potentially missing certain cases, and leading to an uncontrolled reporting bias), the lack of laboratory confirmation of the a fraction of the cases especially those that were reported prior to 1940’s, the lack of genetic information that prevents discriminating between *B*. *anthracis* strains [40], and the absence of information on neighboring states or countries in the analysis. Moreover, the recordkeeping of historic cases inevitably varies by country. Therefore, the findings presented here should be interpreted in light of these limitations. We recognize the importance of genetic diversity in determining Anthrax spread. Yet, the comparison made here may still be useful given that Anthrax strains are relatively homogenous within affected regions [40, 41] and both the Eurasian and North American sub-lineages belongs to the major lineage-A and the two sub-lineages are closely related to each other [40].

It is important to understand that, while scan statistics enables the detection of spatiotemporal clustering patterns, the choice of space-time window parameters was determined based on the sensitivity analysis and the outbreak definition to enable comparison between sites. Resulting cluster sizes are invariably depended on the assigned window parameters. One of the three clusters detected with the 5% space-time parameter setting in northwestern Minnesota clusters indicated a known outbreak of Anthrax occurred in 2006. However, the 5% space-time setting detected two clusters of <1 km radii within a time span of a year in southeastern Kazakhstan, which did not indicate meaningful outbreaks according to the outbreak definition. Therefore, we encourage the value of sensitivity analysis when making the choice of window parameter sizes to optimize detection of outbreaks and enable comparison between sites.

Through this analysis, we have demonstrated that spatiotemporal patterns of anthrax occurrence in two endemic regions may be attributable to specific trigger events. Events such as river floods contributing to the spread of the pathogen and presence of disease hot spots were found at both the sites. However, there were also considerably different factors involved in shaping the spatiotemporal patterns attributable to the site-specific environmental, demographic, and agroecological changes driving occurrence of Anthrax in endemic settings, despite the expected similarities of the pathogen’s behavior relative to the environmental characteristics. Ultimately, results suggest that it is important to consider the probability of recurrence, locations of disease hot spots, and the extents of climatic, environmental, agricultural, and demographic trigger events, both over space and time, when planning strategies for the prevention and control of the disease in endemic settings.

## Supporting information

S1 Table Summary of the historic events in Minnesota related to the spatiotemporal clusters of Anthrax. Key trigger events, i.e. climatic, anthropogenic, agricultural, and environmental changes, which may have led to the recognized clusters are listed.
(DOCX)

S2 Table Summary of the historic events in Kazakhstan related to the spatiotemporal clusters of Anthrax. Key trigger events, i.e. climatic, anthropogenic, agricultural, and environmental changes, which may have led to the recognized clusters are listed.
(DOCX)

S1 Fig Sensitivity analysis of the maximum spatiotemporal window size parameters used in the space-time permutation model of scan statistics. **S**ensitivity analysis was performed using maximum spatial window sizes of 5%, 10%, 20%, 30%, and 50% while the maximum temporal window sizes were held at 5% and 10%. The average cluster sizes in spatial radii (km) and time (years) are illustrated.
(TIF)

S2 Fig Frequency distribution of the spatiotemporal cluster radii of historic animal Anthrax in Minnesota, USA and the Republic of Kazakhstan. Spatiotemporal clusters were detected using the space-time permutation model of the spatial scan statistic with the spatial- and time-windows set to 10% and 5%, respectively.
(TIF)
