# Supplementary material for: Comparison of spatiotemporal patterns of historic natural Anthrax outbreaks in Minnesota and Kazakhstan
Source: PLoS One. 2019 May 17;14(5):e0217144. doi: 10.1371/journal.pone.0217144 (PMC6524940; doi:10.1371/journal.pone.0217144)
Supplement: S1 Table — Key trigger events, i.e. climatic, anthropogenic, agricultural, and environmental changes, which may have led to the recognized clusters are listed. (DOCX) [file pone.0217144.s001.docx]

**S1Table**.

| Potential risk factors and trigger event/s | Reference | #MN-1 | #MN-2 | #MN-3 | #MN-4 | #MN-5 | #MN-6 | #MN-7 | #MN-8 | #MN-9 | #MN-10 | #MN-11 | #MN-12 | #MN-13 |
| --- | --- | --- | --- | --- | --- | --- | --- | --- | --- | --- | --- | --- | --- | --- |
|  |  | 1919-1919 | 1923-1924 | 1925-1926 | 1931-1932 | 1932-1933 | 1933-1933 | 1937-1938 | 1938-1939 | 1945-1946 | 1948-1948 | 1953-1953 | 1996-1997 | 2001-2001 |
| 1. **Favorable soils** | [6] | X | X | X | X | X |  | X | X |  |  | X | X | X |
| 1. **Livestock populations:** Intensive beef cattle production in the southern prairies in early to mid-1900's. | [26] | X | X | X | X | X | X | X | X | X | X | X | X |  |
| 1. **River floods**: Red river flood in April 1997 | [17] |  |  |  |  |  |  |  |  |  |  |  |  | X |
| 1. **River floods**: Minnesota river flood in 1919 | [23] | X |  |  |  |  |  |  |  |  |  |  |  |  |
| 1. **Outbreaks in neighboring states/areas**: Anthrax in wild herbivores in North Dakota duringlate1990's | [6, 18, 19] |  |  |  |  |  |  |  |  |  |  |  |  | X |
