# Supplementary material for: Comparison of spatiotemporal patterns of historic natural Anthrax outbreaks in Minnesota and Kazakhstan
Source: PLoS One. 2019 May 17;14(5):e0217144. doi: 10.1371/journal.pone.0217144 (PMC6524940; doi:10.1371/journal.pone.0217144)
Supplement: S2 Table — Key trigger events, i.e. climatic, anthropogenic, agricultural, and environmental changes, which may have led to the recognized clusters are listed. (DOCX) [file pone.0217144.s002.docx]

**S2 Table**.

| Potential risk factors and trigger event/s | References | #KZ-1 | #KZ-2 | #KZ-3 | #KZ-4 | #KZ-5 | #KZ-6 | #KZ-7 | #KZ-8 | #KZ-9 | #KZ-10 | #KZ-11 | #KZ-12 | #KZ-13 | #KZ-14 | #KZ-15 | #KZ-16 | #KZ-17 |
| --- | --- | --- | --- | --- | --- | --- | --- | --- | --- | --- | --- | --- | --- | --- | --- | --- | --- | --- |
|  |  | 1934-1937 | 1935-1938 | 1940-1940 | 1944-1947 | 1945-1947 | 1949-1952 | 1951-1954 | 1954-1957 | 1956-1958 | 1956-1959 | 1968-1969 | 1971-1971 | 1981-1983 | 1982-1983 | 1985-1987 | 1989-1992 | 2000-2001 |
| 1. **Vaccine coverage**: Insufficient or absent vaccination of susceptible species | [7, 13] | X | X | X | X | X | X | X | X | X | X | X | X |  |  |  |  |  |
| 1. **Livestock populations:** Sharp growth of livestock population | [7, 13] | X | X | X | X | X | X | X | X | X | X | X | X | X | X | X | X |  |
| 1. **Favorable soils**: chernozems and kastanozem | [7] | X | X | X | X |  | X | X | X |  | X | X | X | X | X | X | X | X |
| 1. **Land Use**: Virgin Lands Campaign (between 1954 and 1963) | [31] |  |  |  |  |  |  | X | X | X | X | X | X |  |  |  |  |  |
| 1. **River flooding:** Syrdarya river floods | [7, 13] |  |  |  |  | X |  |  |  | X |  |  |  |  |  |  |  |  |
| 1. **Intensive agriculture** | [32] | X |  |  |  | X |  |  |  | X |  |  | X |  |  | X | X | X |
