# Supplementary figures and images for: Comparison of spatiotemporal patterns of historic natural Anthrax outbreaks in Minnesota and Kazakhstan
Source: PLoS One. 2019 May 17;14(5):e0217144. doi: 10.1371/journal.pone.0217144 (PMC6524940; doi:10.1371/journal.pone.0217144)

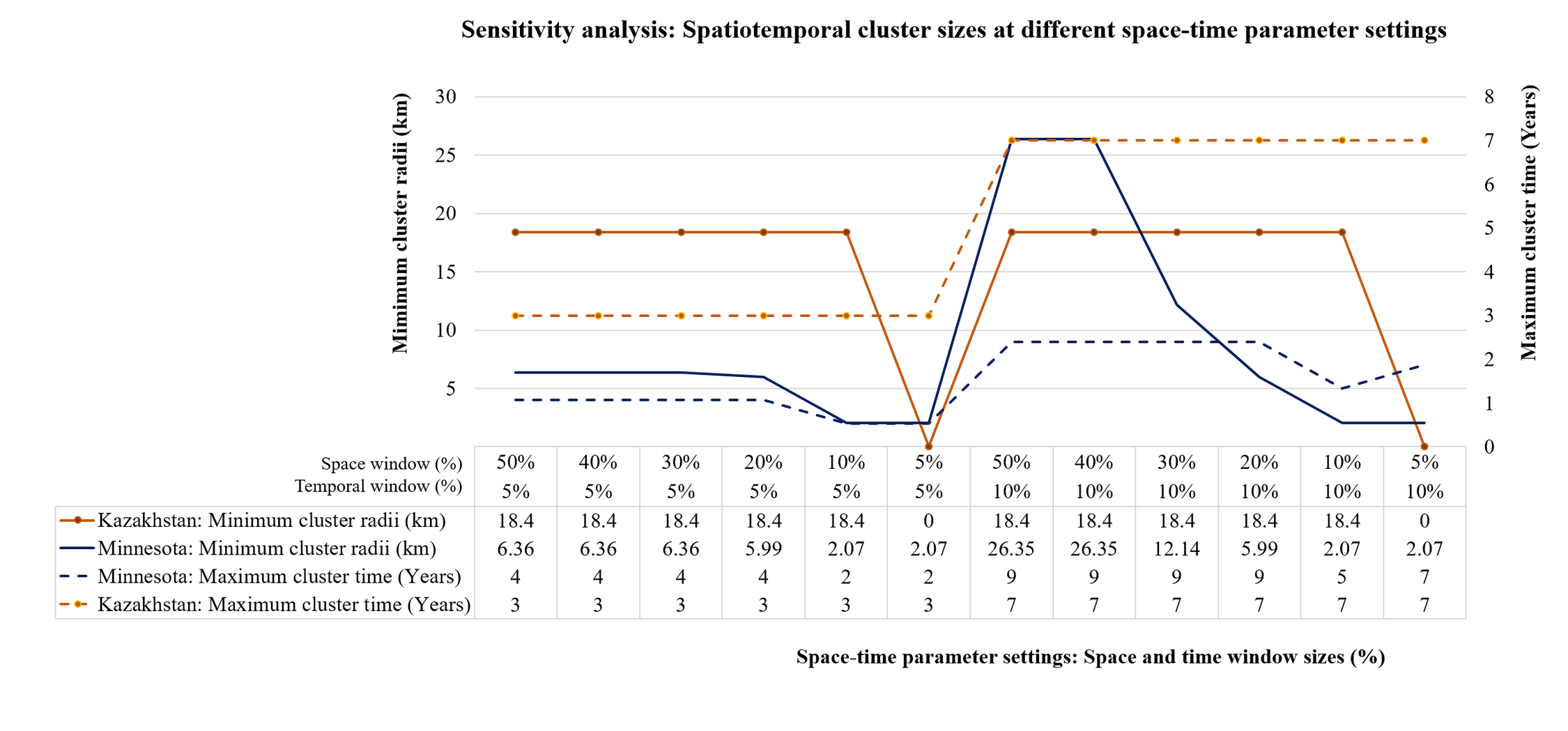

Supplement: S1 Fig — Sensitivity analysis was performed using maximum spatial window sizes of 5%, 10%, 20%, 30%, and 50% while the maximum temporal window sizes were held at 5% and 10%. The average cluster sizes in spatial radii (km) and time (years) are illustrated. (TIF) [file pone.0217144.s003.tif]

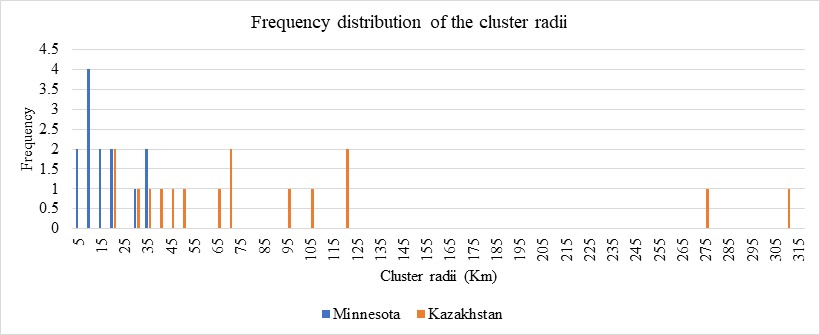

Supplement: S2 Fig — Spatiotemporal clusters were detected using the space-time permutation model of the spatial scan statistic with the spatial- and time-windows set to 10% and 5%, respectively. (TIF) [file pone.0217144.s004.tif]
